# Expression level of long non-coding RNA colon adenocarcinoma hypermethylated serves as a novel prognostic biomarker in patients with thyroid carcinoma

**DOI:** 10.1042/BSR20210284

**Published:** 2021-04-16

**Authors:** Yong Xiao, Youbing Tu, Yuantao Li

**Affiliations:** 1 Department of Anesthesiology, Affiliated Shenzhen Maternity & Child Healthcare Hospital, Southern Medical University, Shenzhen 518028, Guangdong Province, People’s Republic of China; 2 Department of Anesthesiology, Shenzhen People’s Hospital, Shenzhen 518020, Guangdong Province, People’s Republic of China

**Keywords:** colorectal adenocarcinoma hypermethylated, molecular mechanism, overall survival, The Cancer Genome Atlas, thyroid carcinoma

## Abstract

The present study attempts to identify the prognostic value and potential mechanism of action of colorectal adenocarcinoma hypermethylated (CAHM) in thyroid carcinoma (THCA) by using the RNA sequencing (RNA-seq) dataset from The Cancer Genome Atlas (TCGA). The functional mechanism of CAHM was explored by using RNA-seq dataset and multiple functional enrichment analysis approaches. Connectivity map (CMap) online analysis tool was also used to predict CAHM targeted drugs. Survival analysis suggests that THCA patients with high CAHM expression have lower risk of death than the low CAHM expression (log-rank *P*=0.022, adjusted *P*=0.011, HR = 0.187, 95% confidence interval (CI) = 0.051–0.685). Functional enrichment of CAHM co-expression genes suggests that CAHM may play a role in the following biological processes: DNA repair, cell adhesion, DNA replication, vascular endothelial growth factor receptor, Erb-B2 receptor tyrosine kinase 2, ErbB and thyroid hormone signaling pathways. Functional enrichment of differentially expressed genes (DEGs) between low- and high-CAHM phenotype suggests that different CAHM expression levels may have the following differences in biological processes in THCA: cell adhesion, cell proliferation, extracellular signal-regulated kinase (ERK) 1 (ERK1) and ERK2 cascade, G-protein coupled receptor, chemokine and phosphatidylinositol-3-kinase-Akt signaling pathways. Connectivity map have identified five drugs (levobunolol, NU-1025, quipazine, anisomycin and sulfathiazole) for CAHM targeted therapy in THCA. Gene set enrichment analysis (GSEA) suggest that low CAHM phenotype were notably enriched in p53, nuclear factor κB, Janus kinase-signal transducer and activators of transcription, tumor necrosis factor, epidermal growth factor receptor and other signaling pathways. In the present study, we have identified that CAHM may serve as novel prognostic biomarkers for predicting overall survival (OS) in patients with THCA.

## Background

Thyroid carcinoma (THCA) is the high-incidence malignant cancer of the thyroid, which is a malignant cancer derived from thyroid epithelial cells [1]. Compared with other cancers, thyroid cancer has a relatively favorable prognosis except undifferentiated cancer type, and there are many factors that affect the prognosis, such as the age of the patient and genetic variation. Most of THCA prognosis is related to the combined effects of the following factors: pathological type, degree of development of the lesion, age and genetic factors etc [1–3]. Long non-coding RNA (lncRNA) is a non-coding RNA with length greater than 200 nucleotides. Studies have shown that lncRNA plays an indispensable role in multiple life activities such as dose compensation effect, epigenetic regulation, cell cycle regulation and cell differentiation regulation, and has become a hot spot in genetics research [4]. Under normal circumstances, lncRNA may exhibit tumor suppression or carcinogenic functions, when its dysregulation in cancers can promote tumorigenesis and cancer metastasis [5–7]. LncRNA has significant correlation with cancer cell drug resistance, cancer diagnosis and prognosis, and can be used as cancer-related biomarkers [8,9]. Due to the dysregulation of lncRNA in cancer cells play an indispensable role in the growth and differentiation [10]. Therefore, the relationship between lncRNA and cancers are closely related, and are worth further study. Colorectal adenocarcinoma hypermethylated (CAHM) is an lncRNA that is frequently hypermethylated in colorectal cancer (CRC) tumor tissues and its expression is usually down-regulated [11]. So far, the mechanism of CAHM involvement in tumorigenesis and cancer development is still not very clear, and study of CAHM in THCA has not been reported. Because The Cancer Genome Atlas (TCGA) aggregates a large number of genome-wide multi-omics sequencing datasets of common tumors and complete clinical prognostic dataset, it can be used to explore the functional mechanism and clinical application of specific genes [12]. Therefore, the present study attempts to identify the prognostic value and potential mechanism of action of CAHM in THCA by using the RNA sequencing (RNA-seq) dataset from TCGA.

## Materials and methods

### Data downloading and preprocessing

A total of 568 samples’ RNA-seq datasets from 502 patients were obtained from the official website of TCGA, among which 58 were para-carcinoma tissue samples and 510 were tumor tissue samples [13]. There were 507 patients with clinical parameters obtained from TCGA official website. By comparing the clinical parameters and RNA-seq datasets, we obtained 501 THCA patients both with clinical parameters and RNA-seq dataset, and then included them in the subsequent prognosis analysis. RNA-seq dataset was preprocessed with *edgeR* [14]. The datasets used in the present study are all obtained from TCGA, and the data acquisition and use followed the TCGA publication guidelines. Since the authors of the present study were not involved in any animal or human experiments, no additional ethical approval was required.

### Clinically significant investigation of CAHM in THCA

We obtained the expression distribution of CAHM in TCGA pan-cancer cohort through gene expression profiling interactive analysis network (GEPIA: http://gepia.cancer-pku.cn/index.html) [15]. At the same time, the expression distribution of CAHM in cancer and non-cancer tissues of THCA was also compared. We identified the high- and low-CAHM phenotypes in THCA patients for survival analysis based on the median value of CAHM expression. In addition, we also constructed a nomogram containing CAHM expression and clinical parameters for individualized prognostic assessment of THCA patients. Nomogram is generated in the R platform by the *rms* package. At the same time, we also used clinical parameters and CAHM expression to divide THCA patients into multiple subgroups for joint effect survival analysis.

### Functional enrichment of CAHM in THCA

We all know that lncRNAs play a biological role in cancers through the regulation of protein-coding genes. Therefore, we used the THCA RNA-seq dataset to screen for CAHM co-expressed genes. Screening criteria for CAHM co-expressed genes were as follows: Pearson correlation coefficient |r| > 0.4 and *P*<0.05. Through the functional enrichment of these co-expressed genes by Database for Annotation, Visualization, and Integrated Discovery v6.8 (DAVID v6.8, https://david.ncifcrf.gov/home.jsp) [16] and Biological Networks Gene Ontology tool (BiNGO) [17], we can further understand the biological function mechanisms of CAHM involved in THCA. Subsequently, in order to further understand the potential biological mechanisms of prognostic differences in patients with different CAHM expression levels, we also used RNA-seq dataset to screen differentially expressed genes (DEGs) between low- and high-CAHM phenotypes, and explored the underlying mechanisms by functional enrichment of these DEGs. The DEGs screening were performed bythe *edgeR* package in R platform, and the screening criteria for DEGs are as follows: |log_2_ fold change (FC)| > 1, *P*<0.05 and false discovery rate (FDR) < 0.05. Connectivity map (CMap) online tool also used to screening the targeted therapy small molecule compounds for CAHM in THCA [18,19]. The chemical structure and drug–gene interaction network of small molecule compounds were obtained from PubChem (https://pubchem.ncbi.nlm.nih.gov) and STITCH (http://stitch.embl.de) [20], respectively. To further understand which genes in these DEGs are at the core status, we use the weighted gene co-expression network analysis (WGCNA: https://horvath.genetics.ucla.edu/html/CoexpressionNetwork/Rpackages/WGCNA/index.html) method to construct a co-expression interaction network for these DEGs [21,22]. The core genes are identified based on their connectivity with other DEGs. In addition to functional enrichment analysis of DEGs, we also used gene set enrichment analysis (GSEA) to further explore the mechanisms between different CAHM expression levels phenotypes [23,24]. The reference gene sets of GSEA were derived from Explore the Molecular Signatures Database (MSigDB) [25,26]. In this study, the c2 (c2.all.v7.0.symbols.gmt) and c5 (c5.all.v7.0.symbols.gmt) gene sets were used for in-depth mechanism exploration. We considered |normalized enrichment score (NES)| > 1, nominal *P*<0.05 and FDR < 0.25 to be statistically significant.

### Statistical analysis

In the screening of GSEA and DEGs, both were corrected according to FDR method. The Kaplan–Meier survival curves were compared using the log rank test, and univariate and multivariate survival analyses were used Cox proportional hazards regression analysis. Hazard ratio (HR) and 95% confidence interval (CI) were used to compare the risk ratios of survival differences between different subgroups. R platform using version 3.6.2. *P*<0.05 considered the difference to be statistically significant.

## Results

### Clinically significant investigation of CAHM

**Figure 1:**
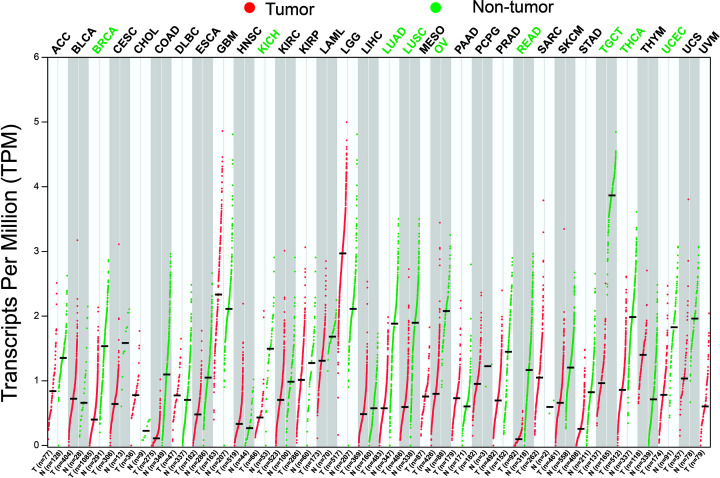
Scatter plot of CAHM expression distribution between tumor and non-tumor tissues in TCGA pan-cancer cohorts

**Figure 2:**
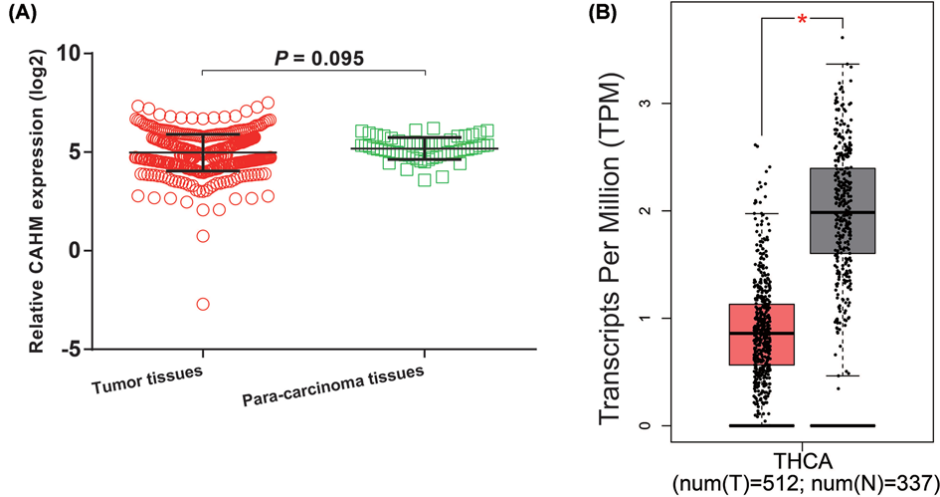
Scatter plot of CAHM expression distribution between tumor and non-tumor tissues in TCGA THCA cohort (**A**) CAHM expression distribution between THCA tumor (*n*=510) and adjacent para-carcinoma tissues (*n*=58). (**B**) CAHM expression distribution between THCA tumor and non-tumor tissues (non-tumor tissues includes paracancerous tissues from the TCGA THCA cohort and normal thyroid tissues from the GTEx database).The red asterisk (*) represents *P*<0.05.

**Figure 3:**
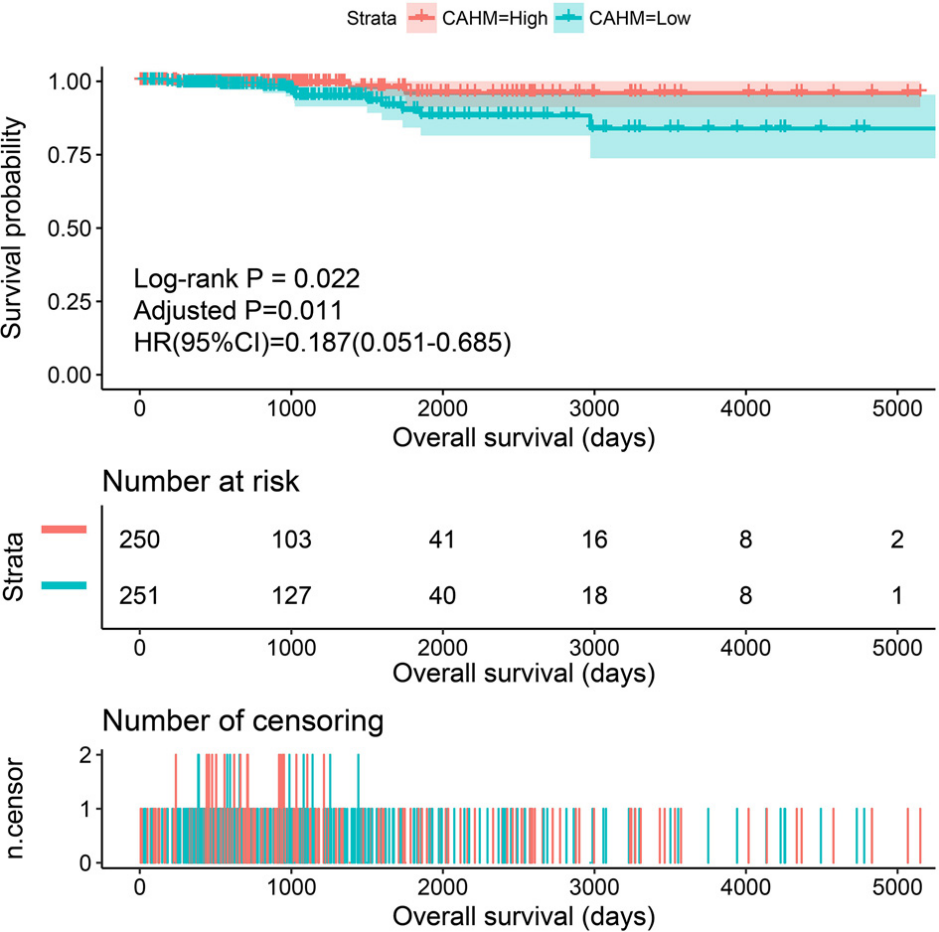
Kaplan–Meier plot of CAHM in TCGA THCA cohort

**Figure 4:**
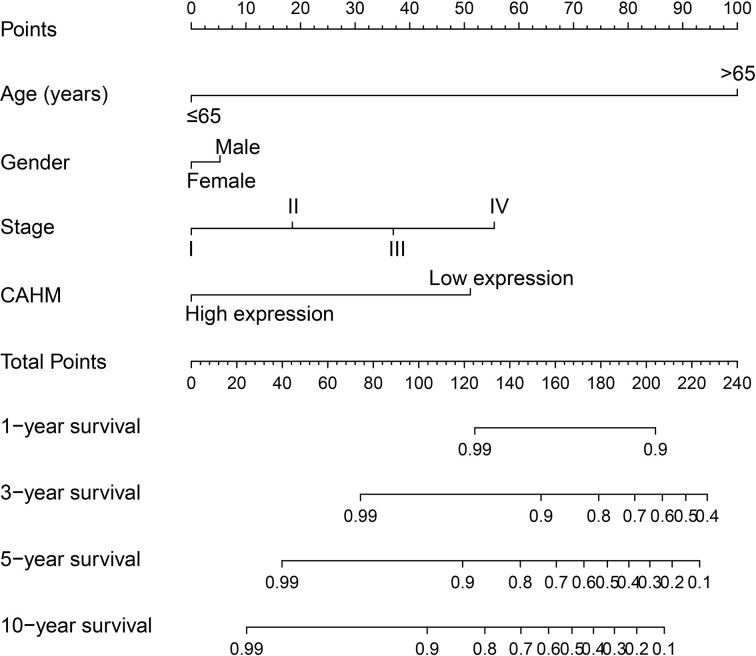
Nomogram of CAHM in TCGA THCA cohort

**Figure 5:**
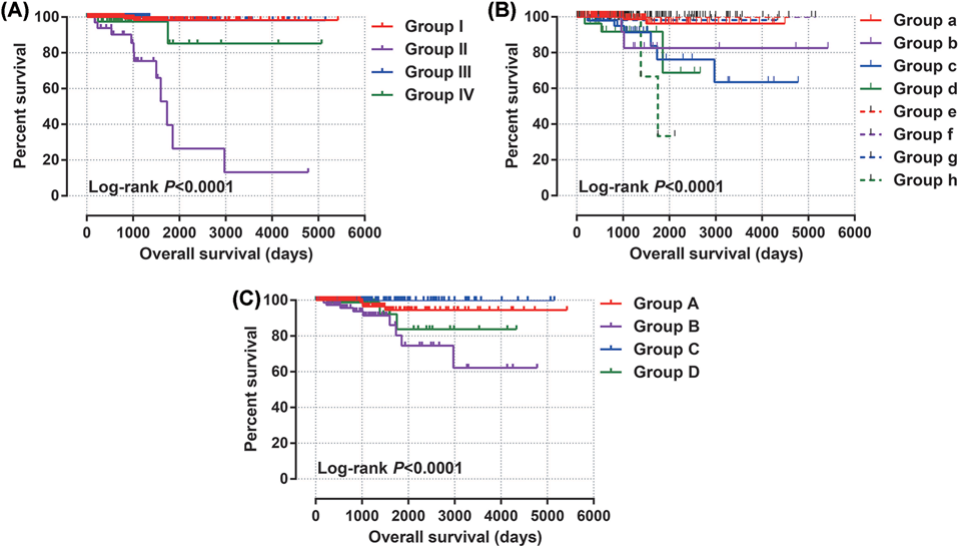
Joint effect survival analysis of CAHM combined with clinical parameters (**A**) Kaplan–Meier plot of CAHM combined with age. (**B,C**) Kaplan–Meier plot of CAHM combined with tumor stage.

By analyzing the expression distribution of CAHM in TCGA pan-cancer datasets, we found that CAHM was significantly down-regulated in tumor tissues of various cancers, including breast invasive carcinoma (BRCA), kidney chromophobe (KICH), lung adenocarcinoma (LUAD), lung squamous cell carcinoma (LUSC), ovarian serous cystadenocarcinoma (OV), acute myeloid leukemia (AML), prostate adenocarcinoma (PRAD), skin cutaneous melanoma (SKCM), stomach adenocarcinoma (STAD), uterine carcinosarcoma (UCS), kidney renal clear cell carcinoma (KIRC), colon adenocarcinoma (COAD), rectum adenocarcinoma (READ), testicular germ cell tumors (TGCTs), uterine corpus endometrial carcinoma (UCEC) and THCA (Figure 1). It is also significantly up-regulated in a variety of tumor tissues, including cholangiocarcinoma (COHL), glioblastoma multiforme (GBM), brain lower grade glioma (LGG), pancreatic adenocarcinoma (PAAD) and thymoma (THYM). The expression distribution of CAHM is different in different cancers, suggesting that CAHM may play the role of oncogene or tumor suppressor gene in different tumors. In THCA tumor and para-carcinoma tissues, we did not observe significant dysregulation of CAHM in THCA tumor tissues (Figure 2A). In comparison with normal thyroid tissues, we found that CAHM was significantly down-regulated in THCA tumor tissues (Figure 2B). The baseline data of 501 THCA patients that included in the prognostic analysis are summarized in Table 1. We found that THCA prognosis was significantly correlated with age and tumor stage. Survival analysis suggests that THCA patients with high CAHM expression have lower risk of death than these with low CAHM expression (log-rank *P*=0.022, adjusted *P*=0.011, HR = 0.187, 95% CI = 0.051–0.685; Figure 3, Table 1). By constructing a nomogram prognostic model, we found that in this cohort, the contribution of CAHM to the death of THCA patients is second only to the age factor with 65 years as the boundary (Figure 4). The effect of CAHM on prognosis of THCA was higher than that of tumor stage. We conducted a joint effect survival analysis of tumor stage and age factors with CAHM expression, and found that high-risk patients can be significantly separated in the Kaplan–Meier survival curves, but after multivariate correction, we did not find that CAHM expression can significantly separate high-risk patients of THCA (Figure 5A–C, Table 2).

**Table 1:** Demographic data of patients in the TCGA thyroid cancer cohort

Variables	Patients (*n*=501)	Crude HR (95% CI)	Crude *P*
**Age (years)**			
≤65	425	1	
>65	76	28.780 (8.179–101.271)	<0.0001
**Gender**			
Female	366	1	
Male	135	1.969 (0.712–5.442)	0.192
Tumor stage*			
Stage I	281	1	
Stage II	52	5.355 (0.749–38.267)	0.094
Stage III	111	9.688 (2.009–46.728)	0.005
Stage IV	55	18.945 (3.637–98.686)	0.0005
Tumor stage*			
Early stage: Stage I+II	333	1	
Advanced stage: Stage III+IV	166	7.190 (2.314–22.345)	0.001
**CAHM**			
Low CAHM	251	1	
High CAHM	250	0.255 (0.073–0.897)	0.033

*Information of tumor stage was unavailable in two patients.

**Table 2:** Joint effect survival analysis of CAHM combined with clinical parameters

Group	CAHM	Variables	Patients (*n*=501)	Crude HR (95% CI)	Crude *P*	Adjusted HR (95% CI)	Adjusted P*
		**Age (years)**					
**I**	**Low CAHM**	**≤65**	218	1		1	
**II**	**Low CAHM**	**>65**	33	45.235 (9.939–205.862)	<0.0001	34.541 (6.745–176.881)	<0.0001
**III**	**High CAHM**	**≤65**	207	0.580 (0.053–6.393)	0.656	0.648 (0.058–7.185)	0.723
**IV**	**High CAHM**	**>65**	43	6.422 (0.903–45.654)	0.063	4.581 (0.595–35.273)	0.144
		**Tumor stage^†^**					
**a**	**Low CAHM**	**Stage I**	149	1		1	
**b**	**Low CAHM**	**Stage II**	22	6.997 (0.981–49.924)	0.052	3.552 (0.470-26.821)	0.219
**c**	**Low CAHM**	**Stage III**	51	8.572 (1.727–42.549)	0.009	1.351 (0.220-8.289)	0.745
**d**	**Low CAHM**	**Stage IV**	28	9.618 (1.596–57.960)	0.014	3.265 (0.476-22.381)	0.228
**e**	**High CAHM**	**Stage I**	132	1.10 × 10^−5^ (3.16 × 10^−206^ to 3.51 × 10^195^)	0.961	7.00 × 10^−6^ (9.17 × 10^−221^ to 5.47 × 10^209^)	0.963
**f**	**High CAHM**	**Stage II**	30	NA	NA	NA	NA
**g**	**High CAHM**	**Stage III**	60	1.564 (0.142–17.271)	0.715	0.266 (0.021–3.395)	0.308
**h**	**High CAHM**	**Stage IV**	27	11.165 (1.544–80.743)	0.017	1.906 (0.228–15.950)	0.552
		**Tumor stage^†^**					
**A**	**Low CAHM**	**Stage I+II**	171	1		1	
**B**	**Low CAHM**	**Stage III+IV**	79	5.070 (1.560–16.475)	0.007	3.891 (0.632–23.963)	0.143
**C**	**High CAHM**	**Stage I+II**	162	7.00 × 10^−6^ (5.44 × 10^−170^ to 9.71 × 10^158^)	0.951	2.00 × 10^−6^ (9.76 × 10^−183^ to 5.56 × 10^170^)	0.95
**D**	**High CAHM**	**Stage III+IV**	87	2.077 (0.463–9.319)	0.34	1.340 (0.176–10.227)	0.778

*Adjusted for age and tumor stage in multivariate Cox risk proportional regression model. ^†^Information of tumor stage was unavailable in two patients. Abbreviation: NA, not available.

### Functional enrichment of CAHM in THCA

**Figure 6:**
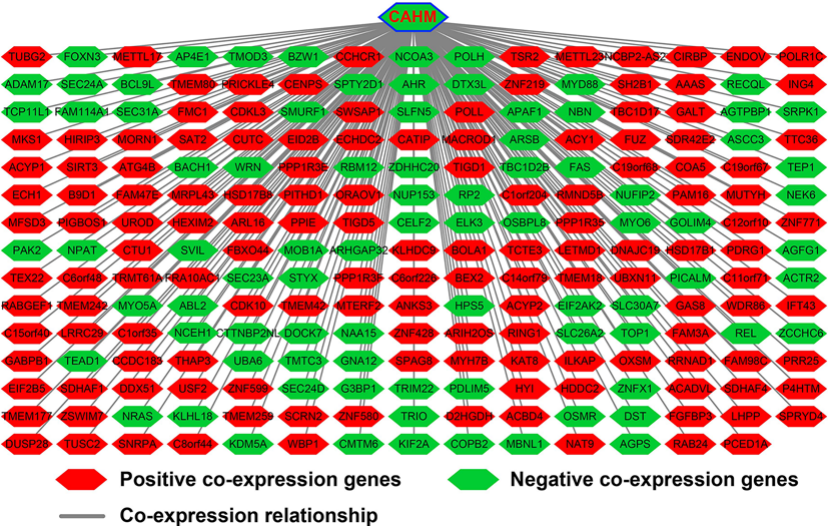
Co-expression interaction networks of CAHM in THCA

**Figure 7:**
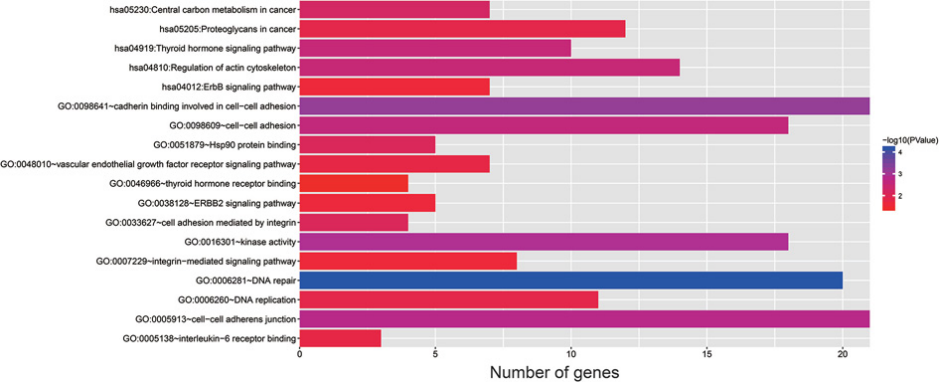
Functional enrichment results of CAHM co-expression genes

**Figure 8:**
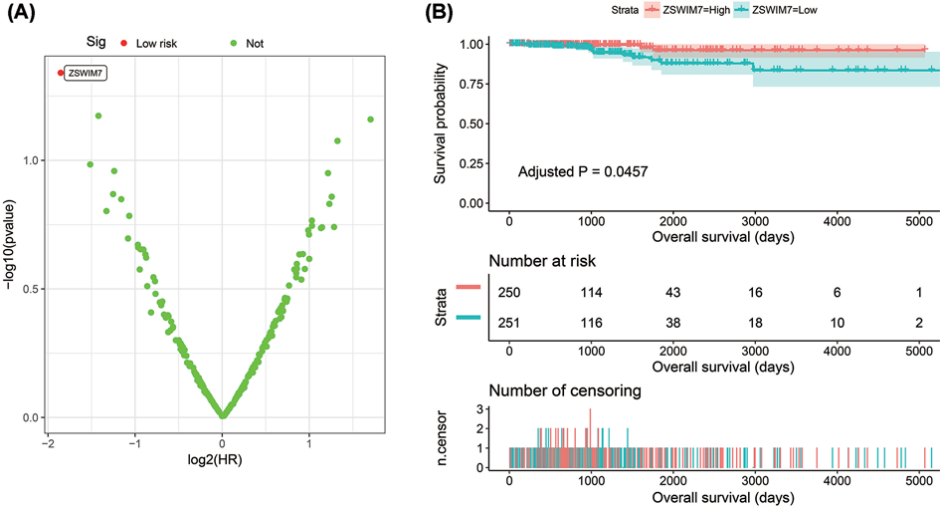
Survival analysis results of CAHM co-expression genes in THCA (**A**) Volcano plot of CAHM co-expression genes survival results. (**B**) Kaplan–Meier plot of ZSWIM7.

Through co-expression analysis, we obtained a total of 224 CAHM co-expressed genes in THCA tumor tissues, of which 84 were negatively co-expressed genes and 140 were positively co-expressed genes (Figure 6, Supplementary Table S1). Functional enrichment suggests that CAHM co-expressed genes are significantly enriched in DNA repair, cadherin binding involved in cell–cell adhesion, cell adhesion mediated by integrin, Hsp90 protein binding, DNA replication, interleukin-6 receptor binding, vascular endothelial growth factor receptor signaling pathway, integrin-mediated signaling pathway, Erb-B2 receptor tyrosine kinase 2, ErbB and thyroid hormone signaling pathways (Figure 7, Supplementary Table S2). Functional enrichment using BiNGO suggest that CAHM co-expressed genes were significantly enriched in response to DNA damage stimulus, cellular response to stress, and cellular metabolic process (Supplementary Figure S1). Subsequently, we also performed prognostic analysis on these CAHM co-expressed genes, and we found that only zinc finger SWIM-type containing 7 (ZSWIM7) among these genes was significantly related to the prognosis of THCA (Figure 8A, Supplementary Table S3). Survival analysis suggests low expression of ZSWIM7 in THCA patients were significantly related to a poor overall survival (OS) (adjusted *P*=0.0457, HR = 0.275, 95% CI = 0.077–0.976, Figure 8B).

**Figure 9:**
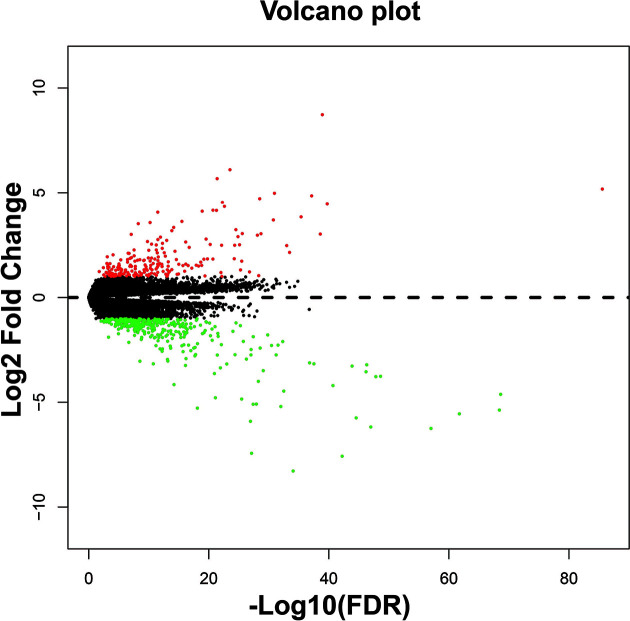
Volcano plot of DEGs between low- and high-CAHM phenotypes THCA patients

**Figure 10:**
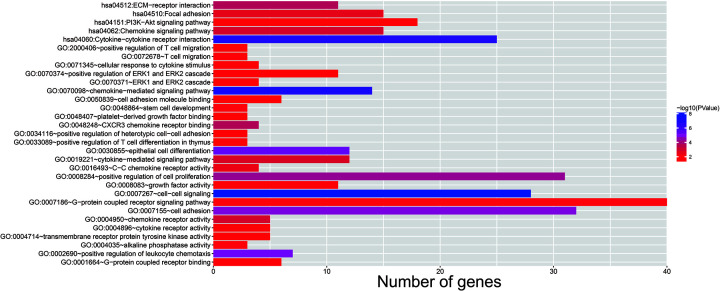
Functional enrichment results of DEGs between low- and high-CAHM phenotypes THCA patients

Using TCGA THCA cohort RNA-seq dataset, we also identified 562 DEGs between high- and low-CAHM expression phenotypes, including 356 down-regulated and 206 up-regulated DEGs (Figure 9, Supplementary Table S4). Heat map of these DEGs were shown in Supplementary Figure S2. Functional enrichment suggest that these DEGs are significantly enriched in cell–cell signaling, chemokine-mediated signaling pathway, positive regulation of cell proliferation, CXCR3 chemokine receptor binding, cytokine-mediated signaling pathway, G-protein coupled receptor signaling pathway, cell adhesion, cellular response to cytokine stimulus, extracellular signal-regulated kinase (ERK) 1 (ERK1) and ERK2 cascade, T-cell migration, positive regulation of T-cell differentiation in thymus, positive regulation of ERK1 and ERK2 cascade, platelet-derived growth factor binding, cytokine–cytokine receptor interaction, extracellular matrix (ECM)–receptor interaction, chemokine signaling pathway, focal adhesion and phosphatidylinositol-3-kinase (PI3K)-Akt signaling pathway (Figure 10, Supplementary Table S5). Functional enrichment using BiNGO also partly supports the above results, suggest that these DEGs were significantly enriched in ECM structural constituent, ECM, cell–substrate adherens junction, regulation of cell differentiation, regulation of immune response, regulation of T cell-mediated cytotoxicity, positive regulation of cell proliferation, cell–cell signaling, positive regulation of cell communication, positive regulation of ERK1 and ERK2 cascade and cell adhesion (Supplementary Figure S3).

**Figure 11:**
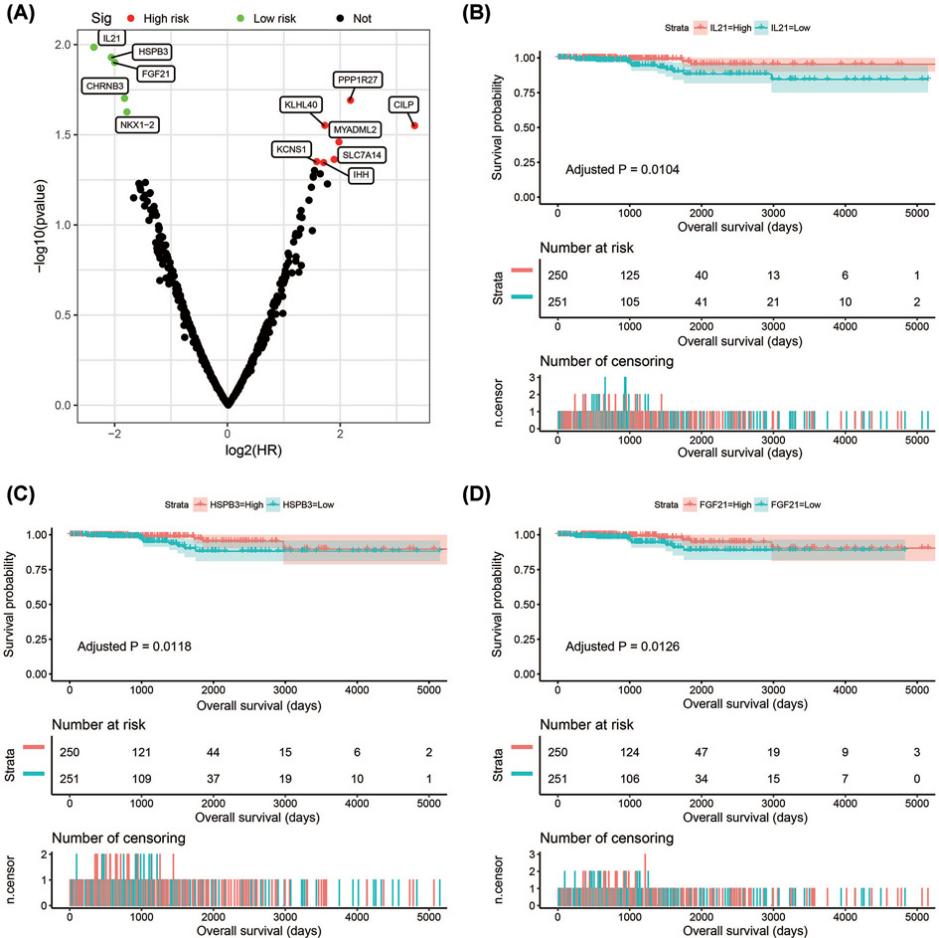
Survival analysis results of DEGs between low- and high-CAHM phenotypes in THCA (**A**) Volcano plot of DEGs survival results in THCA. (**B**) Kaplan–Meier plot of IL21. (**C**) Kaplan–Meier plot of HSPB3. (**D**) Kaplan–Meier plot of FGF21.

Subsequently, we explored the prognostic value of these DEGs in THCA. We used the *survival* package to perform multivariate survival analysis in the R platform, and adjusted the age and tumor stage in the Cox proportional hazard regression model. Through prognostic analysis, we identified 12 DEGs that were significantly related to the prognosis of THCA (Figure 11A, Supplementary Table S6). The top three most significant DEGs were interleukin 21 (IL21: adjusted *P*=0.0104, HR = 0.192, 95% CI = 0.054–0.678, Figure 11B), heat shock protein family B member 3 (HSPB3: adjusted *P*=0.0118, HR = 0.238, 95% CI = 0.078–0.727, Figure 11C) and fibroblast growth factor 21 (FGF21: adjusted *P*=0.0126, HR = 0.248, 95% CI = 0.083–0.741, Figure 11D).

**Figure 12:**
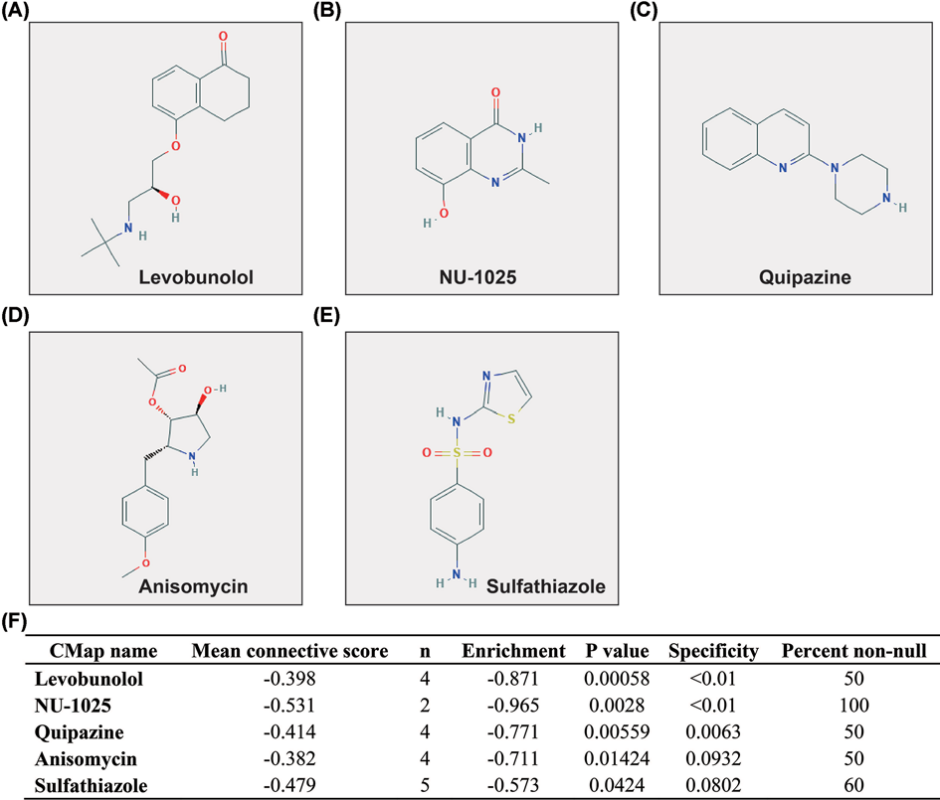
CMap analysis results for low- and high-CAHM phenotypes (**A**) Chemical structure of levobunolol. (**B**) Chemical structure of NU-1025. (**C**) Chemical structure of quipazine. (**D**) Chemical structure of anisomycin. (**E**) Chemical structure of sulfathiazole. (**F**) CMap analysis results.

**Figure 13:**
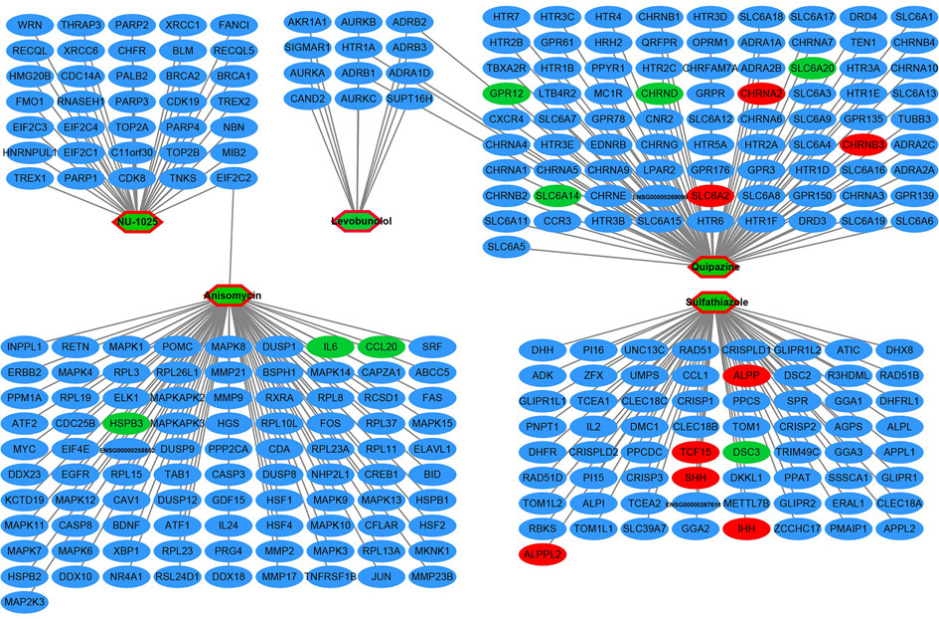
Drug–gene interaction networks generated from STITCH Green nodes represent down-regulated DEGs; red nodes represent up-regulated DEGs; the green prismatic nodes with the red ring represent the drugs; the blue nodes represent the other drug interaction genes.

To screen CAHM’s targeted therapeutic drugs in THCA, we identified five small molecule compounds targeting CAHM in THCA through CMap online tool. The chemical formulas of these five small-molecule compounds are shown in Figure 12A–E, and they are levobunolol, NU-1025, quipazine, anisomycin and sulfathiazole. The detailed analysis results of CMap are shown in Figure 12F. Then we used STITCH to construct the drug–gene interaction networks, and we found that some of these five drugs’ interaction genes were DEGs between different CAHM expression phenotypes (Figure 13). We found that anisomycin can participate in the targeted therapy of CAHM in THCA by regulating HSPB3, interleukin 6 (IL6) and C–C motif chemokine ligand 20 (CCL20), which were DEGs between different CAHM expression phenotypes. While the quipazine is through regulating these DEGs of G protein-coupled receptor 12 (GPR12), solute carrier family 6 member 14 (SLC6A14), cholinergic receptor nicotinic δ subunit (CHRND), solute carrier family 6 member 2 (SLC6A2), cholinergic receptor nicotinic α 2 subunit (CHRNA2), solute carrier family 6 member 20 (SLC6A20), and cholinergic receptor nicotinic β 3 subunit (CHRNB3). In addition, sulfathiazole plays a role in targeting CAHM in THCA by regulating alkaline phosphatase, placental (ALPP), alkaline phosphatase, placental-like 2 (ALPPL2), transcription factor 15 (TCF15), sonic hedgehog signaling molecule (SHH), indian hedgehog signaling molecule (IHH) and desmocollin 3 (DSC3). Among these DEGs, we found that HSPB3, CHRNB3 (adjusted *P*=0.0199, HR = 0.280, 95% CI = 0.096–0.817, Supplementary Table S6), and IHH (adjusted *P*=0.0452, HR = 3.232, 95% CI = 1.026–10.183, Supplementary Table S6) were significantly correlated with the prognosis of THCA. We speculated that anisomycin may function by regulating HSPB3, quipazine may function by regulating CHRNB3, and sulfathiazole may function by regulating IHH, thereby affecting the prognosis of THCA patients with different CAHM expression levels.

**Figure 14:**
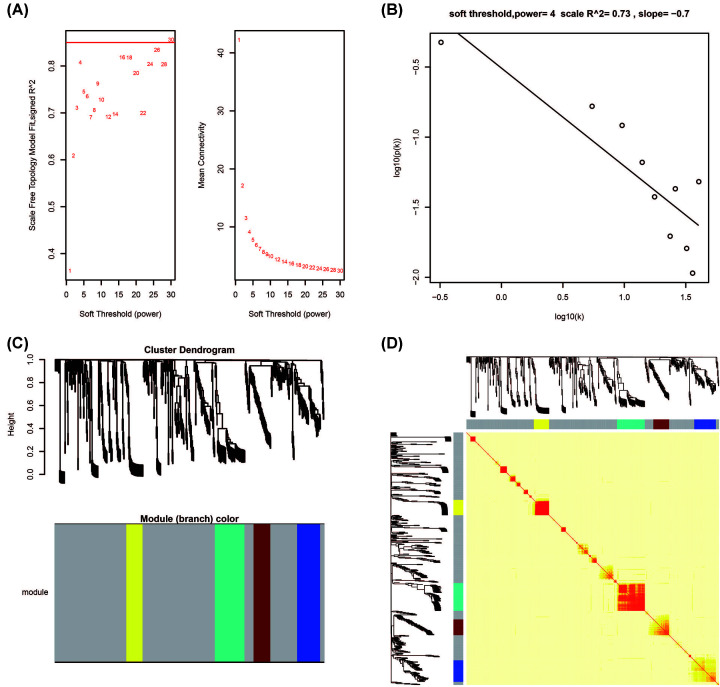
Plot of WGCNA analysis results of DEGs in low- and high-CAHM phenotypes (**A**) Soft threshold screening plot. (**B**) Scale-free topology plot. (**C**) Clustering dendrograms of genes. (**D**) TOM plot.

**Figure 15:**
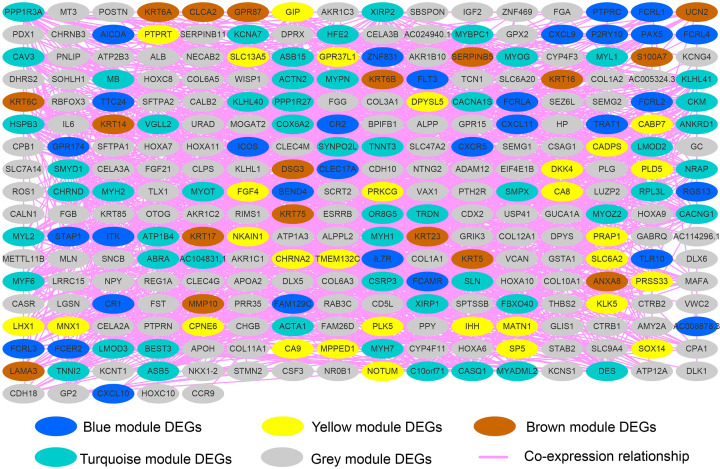
WGCNA networks of DEGs between low- and high-CAHM phenotypes

In order to further understand the interaction between these DEGs, we used WGCNA to construct weighted gene–gene co-expression interaction networks. The appropriate soft threshold power β was set to 4 based on the soft threshold screening (Figure 14A,B), and modular analysis divides these DEGs into five modules of gray, blue, yellow, turquoise and brown (Figure 14C,D). According to the co-expression interaction networks, we identified the top ten genes with the node number as the hub genes of the WGCNA networks (Figure 15). Among the ten hub genes, we found that HSPB3 was significantly associated with THCA prognosis. At the same time, we previously found that it is also one of the top three genes most significantly related to the prognosis of THCA in DEGs, and is significantly related to the targeted therapy of CAHM by anisomycin in THCA. Based on this, we speculate that the effect of this gene is closely related to the prognosis of CAHM in THCA, and it can be used as a biomarker of CAHM in THCA targeted therapy.

**Figure 16:**
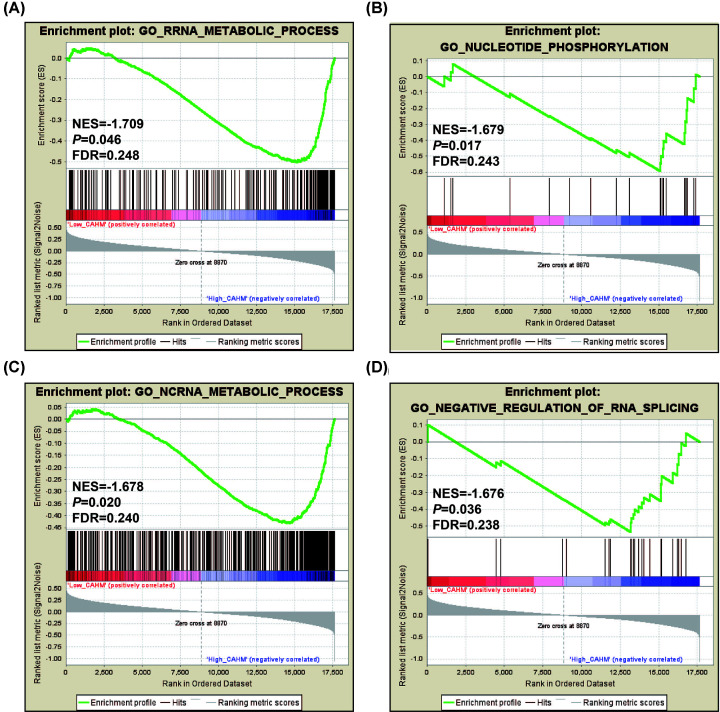
GSEA analysis between low- and high-CAHM phenotypes using the c5 reference gene set (**A**) rRNA metabolic process. (**B**) Nucleotide phosphorylation. (**C**) ncRNA metabolic process. (**D**) Negative regulation of RNA splicing.

**Figure 17:**
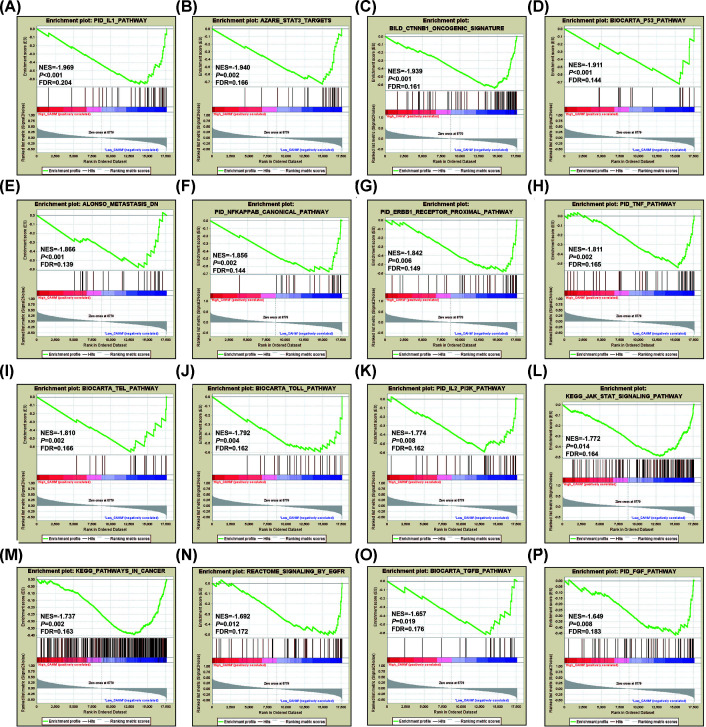
GSEA analysis between low- and high-CAHM phenotypes using the c2 reference gene set (**A**) IL1 pathway. (**B**) STAT3 targets. (**C**) CTNNB1 oncogenic signature. (**D**) P53 pathway. (**E**) Metastasis DN. (**F**) NF-κB canonical pathway. (**G**) ERBB1 receptor proximal pathway. (**H**) TNF pathway. (**I**) TEL pathway. (**J**) TOLL pathway. (**K**) IL2 PI3K pathway. (**L**) JAK STAT signaling pathway. (**M**) Pathways in cancer. (**N**) Signaling by EGFR. (**O**) TGFB pathway. (**P**) FGF pathway.

In addition to DEGs analysis, we also use GSEA to explore the mechanism of CAHM in THCA. Using c5 as a reference gene set, we found that low expression CAHM phenotype can be significantly enriched in RRNA metabolic process, nucleotide phosphorylation, ncRNA metabolic preocess and negative regulation of RNA splicing (Figure 16A–D, Supplementary Table S7). Using c2 as a reference gene set, we found that the low expression CAHM phenotype can be significantly enriched in interleukin 1 (IL1), catenin β 1 (CTNNB1), p53, nuclear factor κB (NF-κB), EebB1 receptor proximal, tumor necrosis factor (TNF), TEL, Toll, interleukin 2 (IL2)/PI3K, Janus kinase-signal transducer and activators of transcription (JAK/STAT), epidermal growth factor receptor (EGFR), transforming growth factor β (TGFB) and fibroblast growth factor (FGF) signaling pathway, signal transducer and activator of transcription 3 (STAT3) targets, metastasis and pathway in cancer (Figure 17A–P, Supplementary Table S8).

## Discussion

Pedersen et al. found that CAHM was hypermethylated in CRC tumor tissues, and its mRNA expression level was significantly down-regulation in CRC tumor tissues, which was negatively correlated with the methylation [11]. By measuring the level of methylated CAHM DNA in plasma, they also found that the positive rate of methylated CAHM DNA was higher in CRC patients than in adenoma or non-cancerous patients. Their findings suggest that both the frequency and amount of methylated CAHM DNA released into plasma increases with the tumor stage of CRC. Methylated CAHM DNA is expected to be a plasma biomarker for CRC screening [11]. Li et al. summarized the triple-negative breast cancer (TNBC) expression profile datasets from the Gene Expression Omnibus database and screened for lncRNA related to the prognosis of TNBC and found that CAHM was significantly related to the prognosis of TNBC. Patients with low CAHM expression had poor prognosis than these with high CAHM expression [27]. Consistent with the results of previous studies, our current study found that the mRNA expression level of THCA was significantly down-regulated in tumor tissues, and THCA patients with low CAHM expression had a shorter OS time than these with high CAHM expression.

Through the enrichment of CAHM co-expressed genes, we found that the functions of these genes are significantly related to the basic growth status of tumor cells such as DNA repair, cell adhesion and DNA replication. In addition, previous studies have shown that cell adhesion plays an important role in tumor metastasis [28,29]. Studies have shown that the expression of HSP90 in medullary THCA is significantly increased, which may be related to the occurrence of THCA, and may become a target of THCA targeted therapy [30]. Inhibition of HSP90 in thyroid cells can significantly increase iodine deposition [31]. Intervention with HSP90 inhibitors in THCA cell lines can significantly inhibit the malignant phenotypes of THCA cells, including inhibiting invasion and inducing apoptosis [32–34]. The content of IL6 in the serum of THCA patients are higher than healthy subjects, and their result suggests that IL6 can be used as a diagnostic marker for THCA. At the same time, both mRNA and protein expression levels of IL6 were up-regulated in THCA tumor tissues by comparing with adjacent normal tissues, and are significantly related to the tumor invasion of THCA. These results indicate that IL6 is closely related to the occurrence and development of THCA [35]. Similar to the above research results, Li et al. also found that single nucleotide polymorphisms of IL6 were significantly correlated with the occurrence and development of THCA, which may be a risk factor for THCA [36]. IL-6 can significantly promote the proliferation and colony formation of THCA stem cells and increase the stem cells and EMT characteristics of THCA. These biological functions of IL6 may contribute to the occurrence and metastasis of THCA [37]. Kunstman et al. detected the tumor tissues of anaplastic THCA by whole exome sequencing and found that most of the mutated genes can be significantly enriched in MAPK and ErbB signaling pathways, indicating that these signaling pathways are significantly associated with anaplastic THCA [38]. They also found that ERBB2 gene mutation was significantly correlated with THCA [38]. The VEGFR signaling pathway plays an essential role in tumor angiogenesis, and drugs that inhibit the VEGFR signaling pathway can be developed for the treatment of THCA [39–41].

Through functional enrichment of the DEGs between the high- and low-CAHM phenotypes THCA, we found that some of these results are closely related to THCA and may have a certain relationship with CAHM affecting the prognosis of THCA. Urra et al. found that CXCR3 was significantly up-regulated in papillary thyroid cancer tumor tissues, and play a role in papillary thyroid cancer oncogenesis [42]. Meanwhile, chemokine-mediated signaling pathway also plays a role in THCA [43]. Multiple studies have shown that drugs or genes can be involved in regulating the proliferation and invasion of THCA and other malignant phenotypes through the ERK1 and ERK2 signaling pathways [44,45]. Similar results can also be found in the PI3K/AKT signaling pathway, genes or drugs through this pathway can significantly regulate the malignant phenotype and disease progression of THCA [46–50].

For the five small molecule compounds targeting CAHM developed in the present study, we found that NU1025 is a poly polymerase inhibitor that can be used to enhance the cytotoxic effects of anticancer drugs [51,52]. Wesierska-Gadek et al. found that NU1025 can play a role in inhibiting proliferation and promoting apoptosis in breast cancer cells by destroying cellular DNA [53]. Recently, multiple studies predicted that NU1025 could be used for the treatment of intracerebral and hepatocellular carcinoma (HCC) through bioinformatics analysis [54,55]. Anisomycin has been reported to have anti-cancer effects in multiple cancers. Aniamycin significantly inhibited the proliferation, invasion, tumorigenic capacity and tumor angiogenesis of human ovarian cancer stem cells [56]. Anithromycin not only plays a direct killing role in HCC, but also plays a role in natural killer cell (NK)-mediated immunotherapy, and may be used as a new drug for HCC treatment [57]. Ushijima et al. found that 5-fluorouracil and anisomycin play a synergies anti-cancer role in CRC [58]. The anticancer effect of anisomycin has been confirmed in osteosarcoma [59], chronic myeloid leukemia [60], renal carcinoma cells [61,62], ehrlich ascites carcinoma [63], diffuse large B-cell lymphoma [64], glioma [65] and CRC [66], but there has been no report on the anticancer effect of anisomycin in THCA. The present study firstly proposed that anisomycin has anticancer effect in THCA by bioinformatics analysis. However, levobunolol, quipazine and sulfathiazole have not been reported to be associated with cancer therapy in previous studies.

According to the analysis results of GSEA functional enrichment, previous studies have found that IL1 can promote the growth of human thyroid cell line NIM 1, but it has an anti-tumor effect in some human THCA differentiation and replication [67,68]. Garcia-Rostan et al. carried out mutation detection on 127 THCA patients’ tumor tissues and found that CTNNB1 mutation was a high-frequency mutation and was significantly associated with poor prognosis of THCA [69]. Previous studies showed that p53 was an independent prognostic factor of THCA, and the clinical outcome of THCA patients with p53 immunohistochemical positive was poor [70,71]. NF-κB signaling pathway plays a indispensable role in cancer cell proliferation, angiogenesis, invasion, metastasis and drug resistance, as well as in THCA [72]. Study have found that targeted inhibition of NF-κB pathway combined with chemotherapy can be used for advanced THCA treatment [73]. For the TNF signaling pathway, Zhang et al. found that TNF-α is higher in the serum of THCA patients than healthy subjects, and the prognosis of patients with high serum TNF-α before surgery is poor [74]. The JAK-STAT signaling pathway is a signaling pathway stimulated by cytokines, and is involved in many important cancer biological processes such as cancer cell proliferation, differentiation, apoptosis and immune regulation [75,76]. Khan et al. found that curcumin through the JAK-STAT signaling pathway can increase the efficacy of chemotherapy for THCA treatment [77]. EGFR signaling pathway plays an important role in physiological processes such as cell growth, proliferation and differentiation, and is closely related to cancer cell proliferation, angiogenesis, tumor invasion, metastasis, apoptosis and prognosis [78,79]. EGFR can also be used for early prognostic risk assessment of THCA [80].

The present study has some limitations that need to be explained. First, the present study belongs to the TCGA single-center study, and lacks a verification cohort to verify its diagnostic and prognostic values.

Secondly, the present study is limited to the enrichment of bioinformatics functions, and lacks the verification of *in vivo* and *in vitro* experiments. Our study is based on 501 THCA patients and more than 10 years of follow-up time. Therefore, our results are reliable. Despite the above research limitations, our research is the first report on the clinical application value and functional mechanism of CAHM in THCA, which can provide theoretical basis and research direction for future study.

## Conclusion

In conclusion, our current study found that CAHM was significantly down-regulated in THCA tumor tissues by using TCGA THCA cohort. THCA patients with low CAHM expression have poorer prognosis than those with higher expression. We also identified five drugs (levobunolol, NU-1025, quipazine, anisomycin and sulfathiazole) for CAHM targeted therapy in THCA. Functional enrichment suggests that CAHM may be involved in cell adhesion, cell proliferation and some classic tumor signaling pathways such as ErbB, PI3K in THCA. The present study is only a preliminary exploration of the clinical significance and functional mechanism of CAHM in THCA, and our findings still need to be further verified in future study.

## Supplementary Material

Supplementary Figures S1-S3

Supplementary Tables S1-S8

## Data Availability

The datasets used during the present study are available from the corresponding author upon reasonable request. The raw RNA-seq dataset of THCA can be obtained from TCGA data portal (https://portal.gdc.cancer.gov/).

## Abbreviations

BiNGO: Biological Networks Gene Ontology tool
CAHM: colorectal adenocarcinoma hypermethylated
CI: confidence interval
CMap: Connectivity map
CRC: colorectal cancer
DEG: differentially expressed gene
ECM: extracellular matrix
ERK: extracellular signal-regulated kinase
FDR: false discovery rate
GSEA: gene set enrichment analysis
HR: hazard ratio
lncRNA: long non-coding RNA
OS: overall survival
PI3K: phosphatidylinositol-3-kinase
RNA-seq: RNA sequencing
TCGA: The Cancer Genome Atlas
THCA: thyroid carcinoma
WGCNA: weighted gene co-expression network analysis
ZSWIM7: zinc finger SWIM-type containing 7
