# Supplementary material for: Expression level of long non-coding RNA colon adenocarcinoma hypermethylated serves as a novel prognostic biomarker in patients with thyroid carcinoma
Source: Biosci Rep. 2021 Apr 16;41(4):BSR20210284. doi: 10.1042/BSR20210284 (PMC8056003; doi:10.1042/BSR20210284)
Supplement: Supplementary Figures S1-S3 [file BSR-2021-0284_supp.pdf]

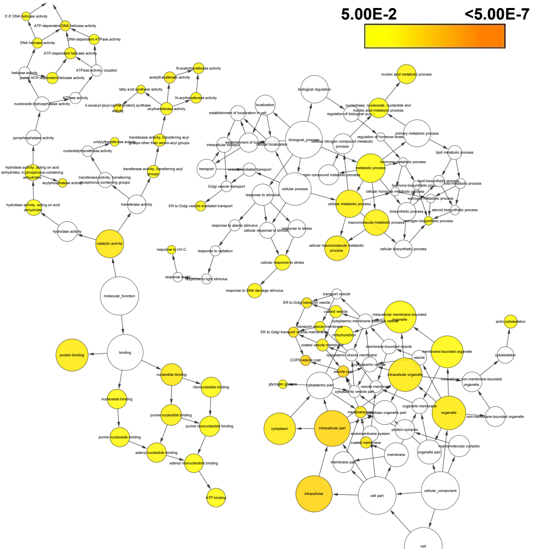

Figure S1. BiNGO analysis results of CAHM co-expression genes.

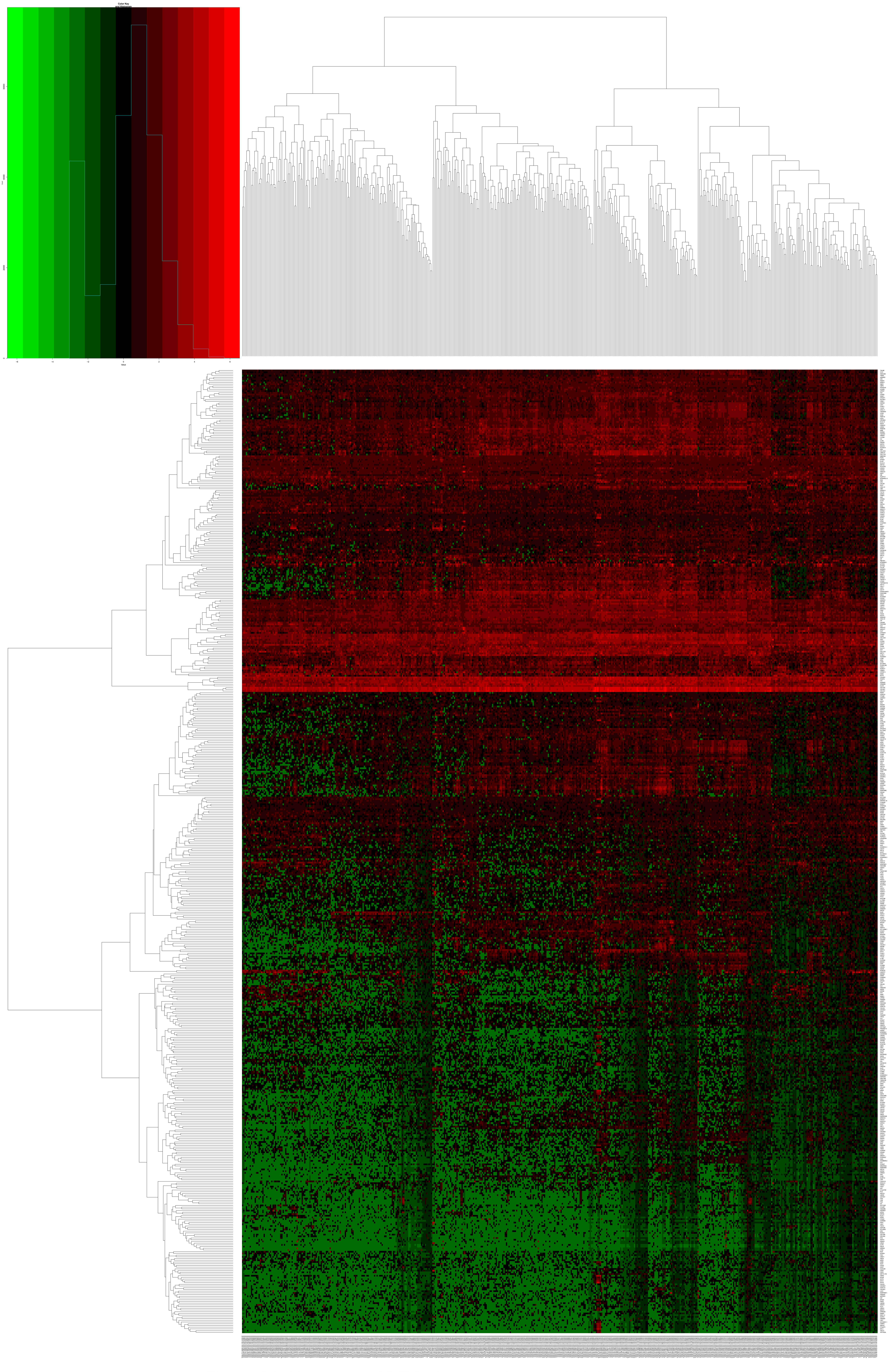

Figure S2. Heat map of DEGs between low- and high-CAHM phenotypes.

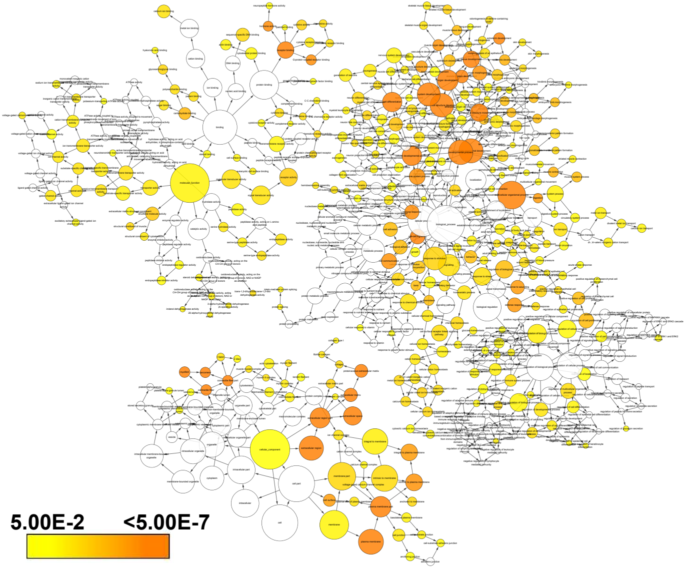

Figure S3. BiNGO analysis results of DEGs between low- and high-CAHM phenotypes.
